# Alternative Splice Variants Modulates Dominant-Negative Function of Helios in T-Cell Leukemia

**DOI:** 10.1371/journal.pone.0163328

**Published:** 2016-09-28

**Authors:** Shaorong Zhao, Wei Liu, Yinghui Li, Pengjiang Liu, Shufang Li, Daolei Dou, Yue Wang, Rongcun Yang, Rong Xiang, Feifei Liu

**Affiliations:** 1 Department of Immunology, School of Medicine, Nankai University, Tianjin 300071, China; 2 Tianjin Entry-Exit Inspection and Quarantine Bureau, Tianjin 300308, China; 3 Department of Hematology, First-Central Hospital, Tianjin 300060, China; 4 State Key Laboratory of Medical Chemical Biology, Tianjin 300070, China; 5 Tianjin Key Laboratory of Tumor Microenvironment and Neurovascular Regulation, Tianjin 300071, China; Pennsylvania State University, UNITED STATES

## Abstract

The molecular defects which lead to multistep incidences of human T-cell leukemia have yet to be identified. The DNA-binding protein Helios (known as IKZF2), a member of the Ikaros family of Krüppel-like zinc-finger proteins, functions pivotally in T-cell differentiation and activation. In this study, we identify three novel short Helios splice variants which are T-cell leukemic specific, and demonstrate their dominant-negative function. We then test the cellular localization of distinct Helios isoforms, as well as their capability to form heterodimer with Ikaros, and the association with complexes comprising histone deacetylase (HDAC). In addition, the ectopic expression of T-cell leukemic Helios isoforms interferes with T-cell proliferation and apoptosis. The gene expression profiling and pathway analysis indicated the enrichment of signaling pathways essential for gene expression, translation, cell cycle checkpoint, and response to DNA damage stimulus. These data indicate the molecular function of Helios to be involved in the leukemogenesis and phenotype of T-cell leukemia, and also reveal Helios deregulation as a novel marker for T-cell leukemia.

## Introduction

The differentiation of hematopoietic stem and progenitor cells into the lymphoid lineage, and the performance of effector activities by lymphocytes at later periods of this developmental pathway rely on the highly organized regulation of transcription factors, which subsequently activate and repress a number of genes [1]. The appropriate function of the immune system depends on the accurate coordination of these pathways, and their deregulations are involved in the pathogenesis of hematopoietic malignancies [2]. Previous studies regarding gene expression in adult T-cell leukemia cells acquired from a diverse number of patients [3] have identified the dysfunction of the Ikaros gene family and the genomic defect of the *Helios* locus [4, 5].

A number of transcription factors play a pivotal role in T- and B-cell development and differentiation, by modulating the expression of downstream targets which regulate the fate decision and activity of lymphoid cells. Ikaros (*IKZF1*) family genes are distinctively expressed in the hematopoietic-lineage cells, and function as the master transcription factor in the control of lymphoid commitment and differentiation [6, 7]. In addition, Ikaros has been recognized as one of the most potent tumor suppressors during leukemogenesis as reported by studies that utilized transgenic mice [8–10]. Recently, several studies have reported the abnormal alternative splicing of Ikaros and the mutation of Ikaros gene locus in human leukemic disorders [11, 12]. These defects are correlated with poor prognoses [13–16]. Subsequent studies have identified the members of Ikaros family as Aiolos (IKZF3), Helios (IKZF2), Eos (IKZF4) and Pegasus (IKZF5) [17, 18]. The members of Ikaros gene family contain up to four N-terminal zinc fingers which mediate the binding to specific DNA motifs (core motif GGGAA), as well as two C-terminal zinc fingers which allow for homo- and hetero-dimerization with other members of Ikaros gene family. Ikaros, Helios and Aiolos are characterized by considerable alternative mRNA splicing of exons, which leads to the production of various isoforms differentially expressed in distinct populations of hematopoietic lineage cells. Isoforms that contain fewer than three N-terminal zinc fingers, are unable to bind DNA but maintain the ability to dimerize, and are recognized to act as dominant-negative factors [8, 19, 20].

Helios is mostly expressed in the T-lineage cells, where it is generally expressed by CD4^+^Foxp3^+^ regulatory T (Treg) and Ly49^+^CD8^+^ Treg lineage cells [19, 21, 22]. Helios was originally proposed to function as a transcription factor that is utilized to identify thymus-derived Treg from peripherally-induced Treg cells. In addition, Helios has been suggested to act as a mediator of T-lymphocyte immune homeostasis, a phenotype of T-lymphocyte differentiation within Foxp3^-^ T helper cells [23], or a marker associated with tolerogenic T-cell immune responses [24–26]. In addition, Helios was found to suppress *IL2* gene transcription by epigenetically silencing Il2 gene expression [27]. More recently, Helios has been postulated to maintain stable inhibitory activity of Tregs cells by activating STAT5 signaling in inflammatory responses [22].

Importantly, genomic alterations and the abnormal expression of Helios have been observed in some patients with T-cell malignancies [4, 25, 28]. Compared to Ikaros, the mechanisms underlying the aberrant Helios expression have yet to be clarified, as a result of limited functional information, which includes the expression patterns and downstream targets of Helios. Therefore, in this study, we performed an investigation regarding the type and function of Helios isoforms, and consequently identified abnormal splicing characteristics, i.e. novel molecular isoforms of Helios, which appear to act as a crucial factor in leukemogenesis leading to abnormal T-cell proliferation.

## Materials and Methods

### Cell lines and clinical samples

Human hematopoietic leukemic cell lines used were listed below: T leukemic cells (CCRF-CEM, MOLT-4, Jurkat), B lymphoma cells (Val, SUDHL4), B lymphoblast cells (HMy2.CIR), myeloid cell lines (HL-60, U937). And non-hematopoietic tumor cell lines were used: breast cancer cell lines (MCF-7, MDA-MB-231), liver cancer cell line (HepG2, SK-Hep1), mesenchymal stem cell (UC10057), embryonic kidney cell (293T), and monkey SV40 transformed kidney fibroblast cell line (COS-7). Normal mesenchymal stem cells were purchased from the China center for Type Culture Collection and were cultured as recommended. Other wild-type cell lines were obtained from the American Type Culture Collection (http://www.ATCC.com) and cultured as suggested by the manufacturer. These cell lines were cultivated in DMEM or RPMI-1640 (Corning) supplemented with 10% fetal calf serum (Hyclone) and 1% penicillin-streptomycin (Gibco). In addition, solvents were added to specific cell lines for survival, such as sodium pyruvate (0.11g/L), 1% L-glutamine, sodium bicarbonate (1.5 g/L), glucose (2.5 g/L) and non-essential amino acids. Peripheral blood mononuclear cells (PBMC) from patients with acute T-cell leukemia and myeloid leukemia were collected with informed consent. Peripheral lymphocytes were isolated by density gradient centrifugation using Ficoll. The project was approved by Research Ethics Committee of Nankai University. The clinical information of leukemic individuals is provided in **Table 1**.

**Table 1:** Clinical characteristics of patients with T-cell leukemia and myeloid leukemia.

No.	Age	Sex	WBC	hemoglobin	platelet	Diagnosis	FACS
	(years)		(*10^9/L)	(g/L)	(*10^9/L)		(% nucleated cells)
1	58	F	13.29	101	230	T-ALL	/
2	12	M	2.65	94	434	T-ALL	T lymphocytes 74.28%
3	62	M	38.09	99	229	T-ALL	/
4	26	F	60.09	69	81	T-ALL	abnormal naïve T lymphocytes, CD7^+^CD10^+^cCD3^+^, 79.88%
5	60	F	39.45	100	114	T-ALL	abnormal T lymphocytes, cCD3^+^CD4^+^CD7^+^, 30.54%
6	28	F	3.22	121	87	T-ALL	abnormal lymphoblastic progenitor cells, CD7^+^CD34^+^CD5^+^ 83.43%
7	49	F	21.3	98	223	CLL	/
8	26	M	6.75	141	140	T-ALL	abnormal naïve T lymphocytes, CD7^+^CD5^+^CD3^+^, 41.77%
9	61	F	3.07	67	4	APL	abnormal myeloid naïve cells, CD117^+^CD64^+^CD33^+^, 96.24%
10	82	F	12.94	53	213	AML-M4b	myeloid naïve monocytes, CD64^+^CD33^+^CD13^+^, 29.43%
11	25	M	20.29	7000	604	AML-M5	abnormal myeloid naïve cells, CD117^+^CD34^+^CD13^+^, 20.04%

Leukemic samples provided from First Central Hospital were used for the expression pattern of Helios isoforms. Clinical information (age, sex, White blood cell count, hemoglobin, platelet, FACS phenotype, and diagnosis) is provided.

### RNA extraction and RT-PCR analysis

Total RNA was prepared from human leukemic cell lines, non-hematopoietic cell lines and mononuclear cells isolated from peripheral blood and bone marrow of patients by using 1ml of Trizol (Invitrogen) per 5×10^6^ cells. The first strand of cDNA was generated with the cDNA synthesis kit (Promega, Madison WI) from total RNA. The amplification of Helios and Ikaros transcripts were carried out by PCR with FastPfu DNA polymerase (Promega). The sense and antisense primers were designed based on exon1 and exon7 of full-length transcript of Helios gene. The nucleotide sequences for primers are listed. Primer1-F: GGATCCATGGAAACAGAGGCTATTGATGGCTA, Primer1-R: GAATTCCTAGTGGAATGTGTGCTCCCCTCGAAC; Primer2-F: GGATCCATGGAAACAGAGGCTATTGAT, Primer2-R: GAATTCAAGGCCTAGTGGAATGTGTG; Primer3-F: GGATCCATGGAAACAGAGGCTATTGATGGC, Primer3-R: GAATTCCTAGTGGAATGTGTGCTCCCCTC. Beta-actin was used as an internal control for cDNA quality.

### Vector construction and sequencing of Helios alternative isoforms

RT-PCR products were separated by electrophoresis in 1.2% agarose gels. DNA bands were excised and purified with QIAquick gel extraction kit (28704; Qiagen). The amplified fragments were directly ligated into the pEASY-Blunt vector (Transgen, Beijing) and sequenced (Sangon Biotech) to verify Helios isoforms. The positive inserts were cloned into pCMV-Tag 2B plasmid labeled with FLAG tag, and then subcloned to pLV-EF1α-MCS-IRES-Bsd expression vector (Cat. #cDNA- pLV 03, Biosettia Inc., San Diego, CA) with FLAG tag.

### Western blotting

Flag-tagged Helios isoforms subcloned in pLV-EF1α-MCS-IRES-Bsd vector were transiently transfected into COS-7 cells with Lipofectamine-2000 (Invitrogen), following standard procedures. The cells (2×10^6^) were harvested, washed with 1 ml PBS, and lysed with 200 μl RIPA buffer (1x phosphate buffered saline, 1% NP-40, 1% SDS, 0.5% sodium deoxycholate, 1 mg/ml aprotinine, 0.1 mM PMSF, 0.1 M Na3VO4) including complete EDTA-free protease inhibitors (Roche) for 30 min on ice. The samples of cell lysate were centrifuged at 16000 g for 10 min at 4°C, and the supernatants were transferred to a new tube for further denaturation. Samples were separated on a 12% SDS-PAGE gel, blotted onto PVDC membrane (0.22 μm pore size) and incubated with specific antibody. The mouse anti-FLAG monoclonal antibody (8146, Cell signaling Technology), anti-Helios (sc-390357 E-7, Santa Cruz; ab168499, Abcam), anti-β actin (Sanjian), and anti-mouse, goat, and rabbit secondary antibodies (Santa Cruz) were used. The membranes were developed by Immobilon ECL immunodetection reagents (Millipore).

### Transient transfection and Immunostaining

COS-7 cells were cultured in 24-well plates with coverslip slides and transfected with pLV-EF1α-Helios-IRES-Bsd expression vectors containing Helios isoforms by Lipofectamine 2000 (Invitrogen). At 48 hr post transfection, Cos-7 cells were subsequently washed three times with 1xPBS, fixed in 4% paraformaldehyde, and permeabilized with 0.5% Triton X-100. The cells were then stained with primary antibodies (diluted with 1% goat serum) with Anti-FLAG (1:1600, 8146; Cell signaling Technology), Anti-HDAC (sc-7872,1:200, Santa Cruz), Anti-MTA2 (sc-28731, 1:200, Santa Cruz), Anti-SIN3A (sc-994, 1:200, Santa Cruz), Anti-Ikaros (H-100, 1:200, Santa Cruz). The Anti-mouse IgG Cy2 conjugated and anti-rabbit IgG Cy3 conjugated secondary antibodies (Jackson immunoResearch) were applied for detection of specific targets, and DAPI (4083; Cell Signaling Technology) was utilized for nuclear staining. The images were acquired by Olympus confocal microscope (Japan).

### Immunoprecipitation

To analyze the interaction of Helios with HDAC, MTA2 or Sin3A in vivo, COS-7 cells which were respectively transfected with Helios isoforms were washed with 1x PBS, and subsequently lysed with 1x RIPA buffer. FLAG-tagged Helios proteins were immunoprecipitated by using a mouse monoclonal anti-FLAG antibody (8146; Cell signaling Technology) and subsequently incubated with protein G-Agarose (CW0012; CWbiotech, Beijing). The agarose was collected and then denatured at 100°C. Samples were loaded onto 8% SDS-PAGE gels. Primary antibodies included Anti-FLAG (8146, 1:1000, Cell signaling), Anti-HDAC (sc-7872, 1:1000, Santa Cruz), Anti-MTA2 (sc-28731, 1:1000, Santa Cruz), Anti-SIN3A (sc-994, 1:1000, Santa Cruz), and Anti-Ikaros (H-100, 1:1000, Santa Cruz).

### Luciferase reporter assay

COS-7 cells were transiently transfected with Helios expression vector and/or Ikaros expression vector by Lipofectamine 2000 reagent. The pGL4.10-firefly vector (Promega) driven by the Hes1 promoter (fragment size 1673 bp) which contained Helios-binding sites at -518 bp and -875 bp was co-transfected as a report vector and RSV-renilla vector was used as an internal control. The luciferase activities were measured by the Dual-Luciferase Reporter Assay System (Promega) after 30 hr transfection.

### Chromatin immunoprecipitation (ChIP) assays

ChIP assay was performed following a procedure provided by a Chromatin IP kit (Beyotime) using the anti-FLAG antibody. A polyclonal antibody against RNA polymerase II (sc-899X; Santa Cruz) was used as a positive control. COS-7 cells were transfected with different Helios isoforms and ChIP was performed after 48h transfection as previously described. The product of DNA was analyzed by using qPCR with specific primer for Foxp3 gene promoter as follows: CHIP-Primer1-F (-1239): CGC CAG CTC TGT GAT CTT G, CHIP-Primer1-R (-1010): TCA GCC TTT GGG ATC TTT; CHIP-Primer2-F (-1006): AAA AGA TCC CAA AGG CTG AGG, CHIP-Primer2-R (-761): TCA TGG AAT CGA AGA GTC GGA G; CHIP-Primer3-F (-715): GCT CTC CAC TTT CTC CTC CAT G, CHIP-Primer3-R (-421): TAA AAT AAA TCA CAG GGC CAA CC.

### Stable transduction

The construction of Jurkat cell lines expressing different Helios isoforms were transducted with lenti-cDNA viral stocks produced by 293T cells. Jurkat cells were plated in 6-well plates with 5×10^5^ cells/well (2.5×10^5^ cells/ml) at the time of transduction. Lentivirus (1ml/well) and 24 μg/well polybrene were added. The plates were centrifuged at 1000×g for 60 min at room temperature. Then transduction medium was gently replaced with 1ml fresh complete medium (repeat this procedure on day 2). Transducted cells (>50% confluence) were selected with 2 μg/ml blasticidin. Finally stable cell lines were cultured to expand for future analysis.

### Apoptosis assays

Cell apoptosis was assessed by use of the Annexin V-FITC Apoptosis Detection Kit (BD Pharmingen). Jurkat cell lines were cultured in 6-well plates with 4×10^5^ cells/ml for 48 hr and then harvested by centrifugation for 5min at 2000rpm. Further staining was processed according to the manufacturer’s protocol. Cisplatin (25 μg/ml) was used to evaluate different drug resistance of Jurkat cell lines stably overexpressing different Helios isoforms.

### Bromodeoxyuridine incorporation assay

Jurkat cell lines were treated with no serum medium for 40 hours and then pulsed with bromodeoxyuridine (BrdU) and complete medium for 3 hours. The number of cells in the S-phase was examined using BrdU Flow Kits (BD Pharmingen). Proliferation was measured with FACS Calibur flow cytometer.

### EdU assay

5-ethynyl-20-deoxyuridine (EdU), a BrdU analog, was incorporated in Jurkat cell lines treated with no serum medium for 2 hr. EdU staining was detected with the Cell-Light EdU Apollo 488 In Vitro Imaging Kit (RiboBio). Stained cells were cytospinned onto coated slides. The proliferation of Jurkat cells was evaluated by fluorescent microscopy. Percentage of EdU positive cells was determined with (EdU add-in cells/Hoechst stained cells) x 100%.

### Gene expression microarray analyses

GeneChip human gene 2.0 ST array (Affymetrix, CA) was used for gene expression microarray. Detailed methods were described previously [9]. Microarray data were deposited in the Gene Expression Omnibus (GEO) database.

## Results

### Identification of novel Helios transcript variants in primary T-leukemic cells

**Fig 1:**
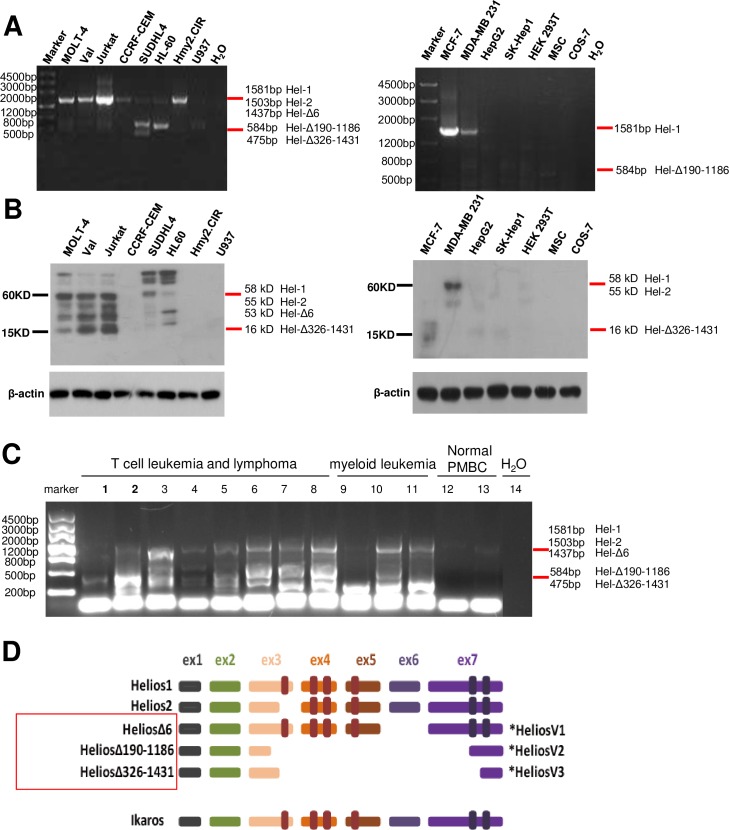
Aberrant expression of Helios transcripts in cell lines and primary T-cell or myeloid leukemic cells. (**A**) RT-PCR to detect Helios expression in hematopoietic cells derived from human T leukemic (Jurkat, CCRF-CEM, MOLT-4), B leukemic (Val, SUDHL4, HMy2.CIR) and myeloid cell lines (HL-60, U937). Samples of non-hematopoietic lineage cells lines derived from breast cancer (MCF-7, MDA-MB 231), hepatic cancer (HepG2, SK-Hep1), renal cancer (HEK 293T), and mesenchymal stem cells (MSC) were included for the test. To identify alternative splicing isoforms, RT-PCR analyses were performed with the primers designed in exons 1 and 7 of canonical full-length Helios transcript. Amplified cDNAs were cloned and sequencing analyses were performed. (**B**) Westernblot analysis of Helios in hematopoietic and non-hematopoietic lineage cell lines to show the presence of multiple Helios isoforms. The molecular weights of Helios short variants were 53 kDa (Helios V1), 10 kDa (Helios-V2) and 16 kDa (Helios-V3). (**C**) Increased expression of Helios short variants in hematological malignancies. RT-PCR analysis of Helios mRNA was performed in peripheral mononuclear blood cells from individuals with acute T-cell-derived leukemia (n = 9) or myeloid leukemia (n = 3), as well as samples from healthy subjects. (**D**) Diagram of Helios-1, Helios-2, and short Helios splice variants identified in this study. Helios variant 1 (V1), variant 2 (V2) and variant 3 (V3) are novel alternative splice isoforms identified in leukemic cell lineages. The brown rectangles in exons 3–5 represent the four N-terminal zinc finger motifs utilized in DNA consensus-sequence binding. The two purple rectangles in exon 7 show the C-terminal zinc finger motifs. Three novel Helios isoforms are nominated according to the exons or nucleotides deleted from the wild-type Helios transcript. The canonical full-length Ikaros 1 is included for comparison.

We first tested the specific expression patterns and levels of Helios in cell lines originated from T- and B-cell lymphoid malignancies, and PBMCs collected from a panel of patients carrying T-cell leukemia or acute myeloid leukemia. In contrast to the control PBMCs from healthy volunteers, leukemic cell lines and primary leukemic cells exhibited noticeable abnormalities in the expression patterns of Helios. RT-PCR and sequencing analysis enabled the characterization of different Helios variants. In addition to the main isoforms of full-length Helios-1 (1581bp) and Helios-2 (1503bp), which lack the part of exon 3 that encodes the first N-terminal zinc finger within the DNA binding domain, we identified three novel hematopoietic-lineage specific Helios short transcripts (Helios-V1, -V2, and -V3) that have not yet been reported (**Fig 1A and 1B**). Helios-V1 (Helios-Δ6, 1437 bp) has all six zinc-finger domains, but lacks exon 6, which possibly contains the nuclear localization signal (NLS) [29] (**Fig 1D** and **S1 Fig**). Helios-V2 (Helios-Δ190–1186, 584bp) and -V3 (Helios-Δ326–1431, 475bp) are non-canonical short splicing variants, as indicated by DNA sequencing (**Fig 1D** and **S1 Fig**). The Helios-V2 and -V3 lack part of exon 3 and all of exon 4–6, which results in the loss of all four N-terminal zinc fingers. In addition, they contain nonsense mutation in exon 7, leading to short coding region sequence and the absence of two C-terminal zinc fingers. In comparison, all Helios isoforms previously described contain the two C-terminal zinc finger motifs [4]. Therefore, it is suggested that Helios-V2 and -V3 are incapable of binding DNA, and possibly function as dominant-negative isoforms [19, 30]. In addition, RT-PCR analysis revealed that these aberrant Helios isoforms were also expressed in some patient samples with T-cell leukemia and myeloid leukemia (9/11 cases) (**Fig 1C and Table 1**). Altogether, our analysis indicated that Helios expression was frequently deregulated in hematopoietic-lineage leukemic cells, and is possibly relevant in the development of hematological malignancies.

In addition, we investigated Helios gene expression by RT-PCR analysis in non-hematopoietic human cells, for example the hepatic cancer cell line HEPG2 and SK-HEP1, breast cancer cell lines MCF7 and MDA-MB231, renal cancer cell line HEK-293T, and mesenchymal stem cell MSCs (**Fig 1A**). Westernblot by anti-Helios antibody was performed to verify the identity of the RT-PCR results (**Fig 1B**). These analyses demonstrated the fact that these non-hematopoietic cells mostly donot express Helios isoforms. Consistent with previous reports, Cos-7 cells were shown to be negative for Helios expression [31].

### Cytoplasmic localization of short Helios isoforms

**Fig 2:**
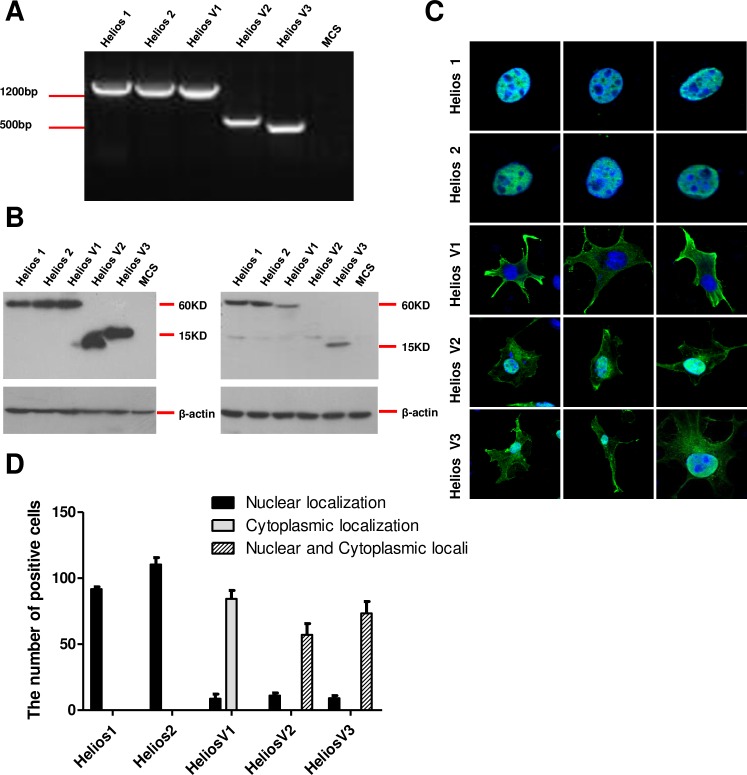
Subcellular distribution of leukemia-type Helios isoforms. (**A, B**) Cos-7 cells were transfected with the indicated five Helios isoforms and subjected to RT-PCR (**A**) and Western blot analyses (**B**). The COS-7 cell extracts were developed with the mouse monoclonal anti-Flag antibody (**left**) and an anti-Helios antibody (**right**). (**C**) Immunofluorescence localization of Helios proteins. The COS-7 cells were transfected with different Helios isoforms. The cellular localization of each protein was visualized with the anti-Flag antibody and then stained with a secondary anti-mouse antibody conjugated with Cy2 (green). The nuclei were counterstained by DAPI staining (blue). Helios-V1, Helios variant 1; Helios-V2, Helios variant 2; Helios-V3, Helios variant 3. (**D**) Graphic representation of the subcellular localization of Helios alternative-splicing variants. The analysis was conducted by counting nuclear or cytoplasmic staining, or both (nuclear and cytoplasmic), of the five isoforms as percent point. Eight fields were counted for each transfection.

To explore the function of Helios isoforms in interacting with each other and modulating gene expression, we cloned five Helios isoforms reciprocally into pLV constructs with FLAG epitope. In addition, for transient transfection, we chose COS-7 cells which do not express either Ikaros or Helios or Aiolos genes [29, 31], and create a system to investigate the effects of individual Helios isoforms without perturbation with endogenous Ikaros-family genes. After transient transfection, the expression of Helios mRNA and proteins in COS-7 cells was validated by RT-PCR (**Fig 2A**) and Western blot (**Fig 2B**), using a monoclonal anti-Flag antibody and an anti-Helios (E-7) antibody. Due to the alternative splicing to generate short variant and the resultant lost of the epitope, Helios-V2 could not be detected with anti-Helios Abs (**Fig 2B**).

The distribution and subcellular localization of the five Helios isoforms was investigated by means of transient transfection in COS-7 cells and by immunofluorescence using the anti-Flag antibody. This experiment showed an isoform-specific cellular distribution in the Helios variants. Ectopically expressed full-length Helios-1 and Helios-2 are localized in the nucleus, but are excluded from the nucleoli [32] (**Fig 2C and 2D**). With regard to the leukemia-type short Helios isoforms, we found that Helios-V1 showed a predominant cytoplasmic localization (**Fig 2C and 2D**), whereas Helios-V2 and -V3, both of which lack exon 3–6, as well as all zinc-finger binding domains, exhibited both cytoplasmic and nuclear staining (**Fig 2C and 2D**). However, none of the dominant-negative short Helios variants showed solely nuclear localization of the protein (**Fig 2D**). In addition, consistent with previous reports [4, 29], exon 6 was shown to be essential for nuclear localization of Helios variants.

### Distinctive isoform-specific interaction with histone deacetylase-containing complexes

Earlier studies have demonstrated that Ikaros-family genes interact with histone deacetylase (HDAC) containing complexes, Mi-2β/NuRD and SIN3 [32, 33], and also associate with SWI/SNF complexes which are ATP-dependent chromatin remodeling complexes [34]. In this study, we tested whether or not Helios isoforms are associated with HDAC-containing complexes. Total cell lysates from Helios transfected Cos-7 cells were used for protein immunoprecipitation with anti-FLAG antibody, and then followed by Westernblot with antibody against HDAC1, which is a histone deacetylase for both Mi-2β/NuRD and SIN3 co-repressor complexes [35]. We also tested by using antibodies against SIN3A and MTA2, the latter of which was found to be a subfraction of the Mi-2β/NuRD complex [36].

**Fig 3:**
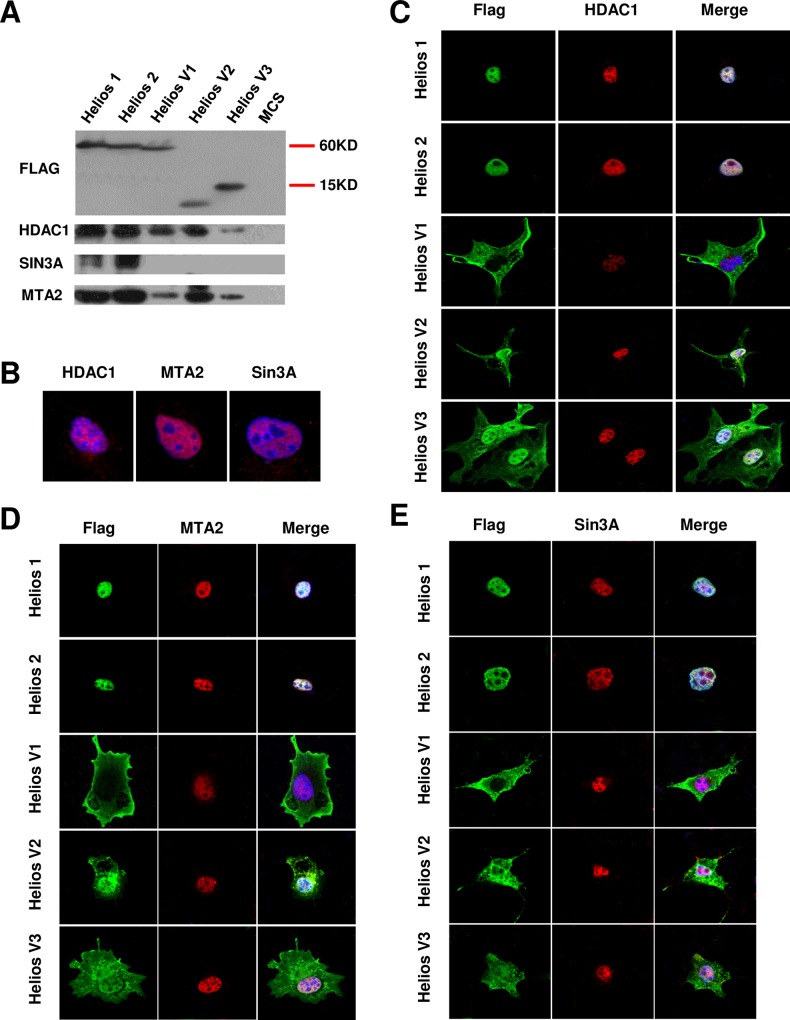
**In vivo interaction between Helios isoforms and histone deacetylase** (A) Association of Helios splice variants and constituents of the Mi-2β/NuRD and SIN3 histone deacetylase complexes. The Helios complexes were immunoprecipitated with an anti-FLAG antibody and the eluates were subjected to Westernblot with anti-FLAG, anti-SIN3A, anti-MTA2, and anti-HDAC1 antibodies which identify the sub-fractions of the Mi-2β/NuRD complex. (**B**) Confocal microscopy of untransfected COS-7 cells to show the nuclear localization of HDAC1, SIN3A and MTA2. (**C-E**) Visualization of COS-7 cells transfected with each Helios isoform by confocal microscopy. Helios splice variants were visualized with an anti-FLAG and a secondary Ab conjugated with Cy2 (green). The proteins HDAC1 (**C**), MTA2 (**D**) and SIN3A (**E**) were stained by antibodies conjugated with Cy3 (red). The nuclei were detected by DAPI staining (blue).

Immunoprecipitation analysis demonstrated direct protein-protein interaction between Helios isoforms and the various chromatin-remodeling factors mentioned above (**Fig 3A**). We observed that both the full-length Helios (1 and 2) and leukemic-type short Helios isoforms (V1-V3) interact with both HDAC1 and the Mi-2β/NuRD subunit MTA2 (**Fig 3A**), while only Helios-1 and -2 appeared to co-immunoprecipitate with SIN3A. Therefore, these results show that Helios recruits the Mi-2β/NuRD and SIN3A histone deacetylase complexes.

In addition, we validated the subcellular co-localization of Helios variants with HDAC1, SIN3 and MTA2 by means of immunocytochemistry. These three factors were shown to homogenously localize in the nuclear of untransfected COS-7 cells (**Fig 3B**). We observed the strong co-localization of the Helios-1 and -2 isoforms with HDAC1, MTA2 and SIN3A (**Fig 3C–3E**). In contrast, the co-localization among Helios-V1 with HDAC1, MTA2 and SIN3A was weak, as anticipated by the protein structures. However, Helios-V2 and -V3, which were distributed in both the cytoplasm and nucleus, were localized with these chromatin-remodeling factors.

### Helios alternative isoforms differentially regulate the promoter of target gene foxp3

**Fig 4:**
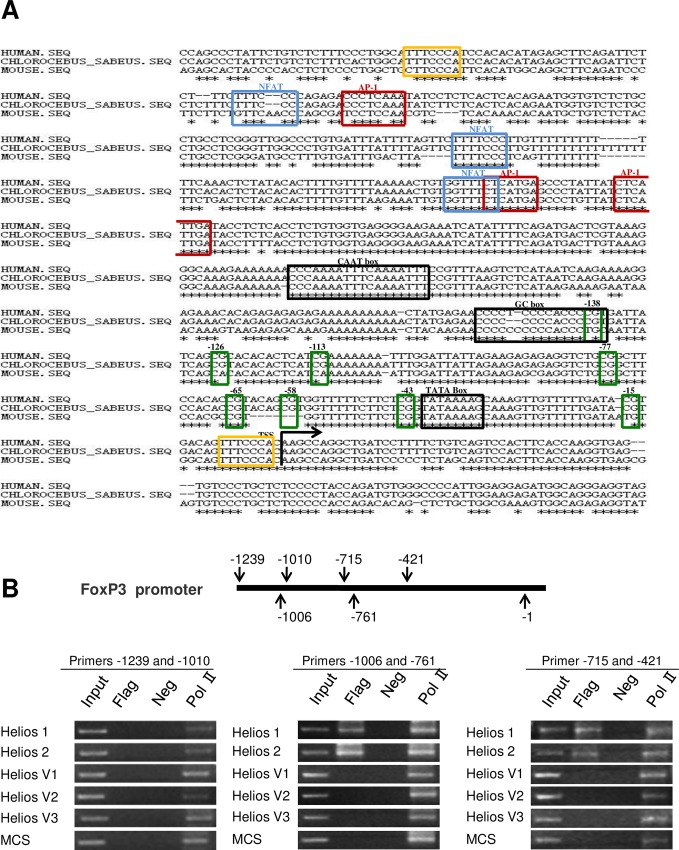
Enrichment of Helios isoforms at the Foxp3 promoter. **(A)** Clustal alignment of human, monkey (COS-7 cells) and mouse genomic sequences showing the conservation of Foxp3 core promoter. The black arrow indicates the location of transcription start site. The locations of the CAAT box, GC box and TATA box are shown by black box. Green boxes illustrate the CpG dinucleotides. The binding sites of transcription factor NF-AT, AP-1 and Helios are marked with red, blue and yellow boxes, respectively. **(B)** ChIP-coupled PCR assay of the enrichment of Helios isoforms at binding motifs at distinct positions on the Foxp3 promoter. ChIP was performed using anti-Flag and anti-RNA polymerase II (positive control) antibodies in COS-7 cells transfected with different Helios isoforms. Negative controls (Neg) are also shown, as are the Input and bound fractions.

Given that Helios is associated with nuclear remodeling complex proteins, we analyzed whether or not chromatin in the area of foxp3 promoter is accessible to the transcription factor Helios variants [37–39]. To do so, we examined the interaction of Helios isoforms with the foxp3 promoter by performing a chromatin immunoprecipitation (ChIP) analysis. Chromatin DNA was immunoprecipitated with anti-Flag antibody, and then tested by PCR with primers for the foxp3 promoters. To design the specific primers we focused on the promoter fragment containing the DNA binding motif for Helios (TGGGAAA) located at promoter region -2bp, -495bp and -828bp [30] (**Fig 4A**). We then observed the specific binding of Helios-1 and -2 to the regulatory region of Foxp3 containing the proximal promoter region (positions -1006 to -421) (**Fig 4B**), which indicated that Helios isoforms potentially function in the transcription regulation of the foxp3 expression. In comparison, ChIP analysis with Helios-V1, which predominantly resides in cytoplasm, was not associated with the foxp3 promoter (**Fig 4B**). As anticipated, Helios-V2 and -V3, which are absent of the DNA-binding zinc finger motifs, did not bind to the foxp3 promoter.

### Short Helios variants display dominant-negative function against wild-type Helios and Ikaros

**Fig 5:**
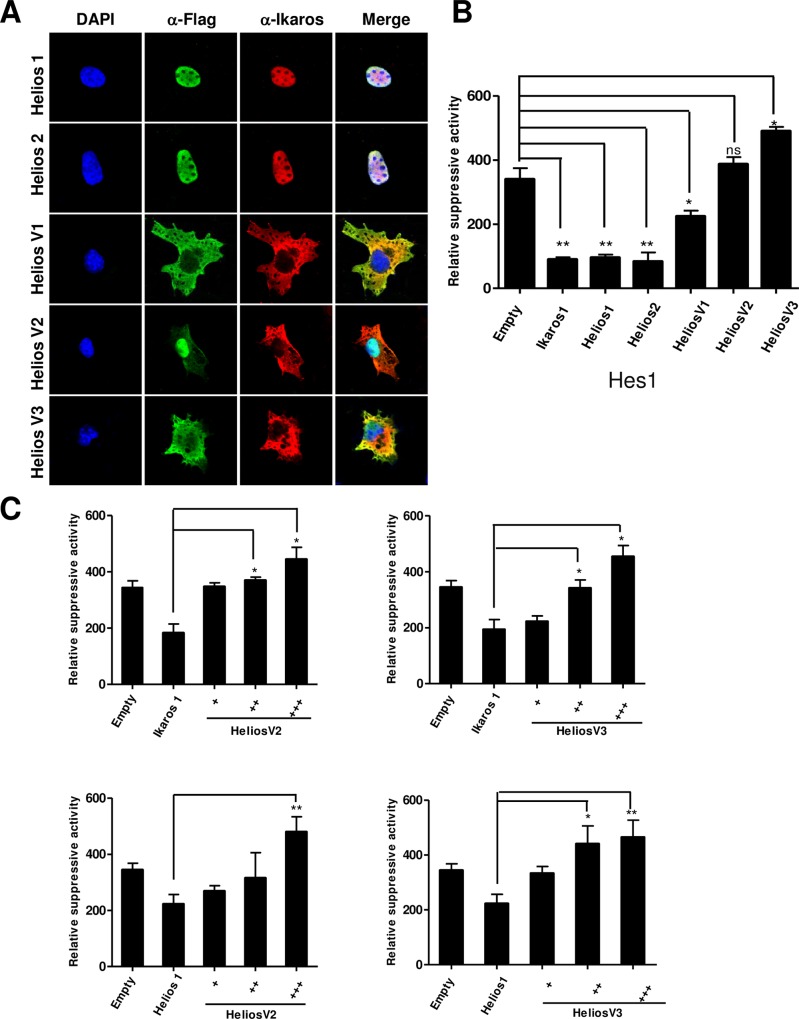
Helios short splice variants exhibit dominant-negative function in the suppressive activities of full-length Ikaros or Helios. (**A**) Co-localization of wild-type Ikaros and leukemia-type short Helios isoforms. COS-7 cells were co-transfected with pLV expression plasmids encoding Ikaros and FLAG-tagged leukemia-type Helios variants. Staining for expressed factors was performed with anti-FLAG or anti-Ikaros antibodies, and visualized with Cy2 (green) and Cy3 (red) respectively. The nucleus was counterstained with DAPI (blue). The samples were analyzed by confocal laser microscopy. (**B**) Suppressive transcriptional activities of individual Ikaros or Helios isoforms demonstrated by *Hes1* promoter dual-luciferase reporter systems. The diagram shows the relative changes in luciferase activity in COS-7 cells co-transfected with promoter reporter construct of Hes1 and with the pLV expression constructs encoding various Helios or Ikaros isoforms. Basal activity of *Hes1* promoter was defined as firefly ⁄ renilla ratio. Data are Mean ± SD, n = 3. (*p < 0.05; **p < 0.01 vs. the control group). (**C**) Functional dominant-negative activity of Helios-V2 and Helios-V3 against Ikaros-1 and Helios-1 determined by *Hes1* promoter assay. The molar ratios of Ikaros-1 or Helios-1 to Helios-V2 and-V3 plasmids are 1:1, 1:2, and 1:3 respectively. Relative luciferase activities were measured as the firefly ⁄ renilla ratio. Each bar indicates the Mean ± SD of three replicates.

Previous research has shown that Helios is a component of multimeric complexes, and is associated with Ikaros in immature T-cells [28, 30]. To examine how the subcellular distribution is modulated by the heterodimer formation, we transfected Cos-7 cells simultaneously with full-length Ikaros and the Helios alternative isoforms. Immunofluorescence and confocal microscopy with anti-Ikaros and anti-Flag antibodies revealed that Ikaros-1, Helios-1 and Helios-2 have identical patterns of nuclear localization (**Fig 5A**). In contrast, Ikaros-1 which was localized exclusively in the nucleus in the absence of Helios [29, 32], when expressed in the presence of Helios-V1, -V2, or -V3, was found to distinctively change its localization from the nucleus into the cytoplasm and co-localize with Helios short variants (**Fig 5A**). Therefore, although Helios-V2 and -V3 lack all six zinc-finger domains, they can still interact and form hetero-dimerization with Ikaros-1. These results suggest that the newly identified Helios isoforms are capable of sequestering and inactivating Ikaros-1. This ability provides the dominant-negative characteristic to the short Helios variants [40], as well as the possible consequent remodeling of the gene expression.

Next, the effects of short Helios variants on the transcriptional control of an Ikaros-regulated Hes1 promoter were analyzed. Indeed, dual-luciferase reporter assay indicated that Helios-1, -2 and Ikaros-1 suppressed *Hes1* promoter activity [4]. Helios-V1, which lacked exon 6 and was localized in cytoplasm, possessed half-suppressive activity against the *Hes1* promoter. In comparison, leukemic-type Helios-V2 and -V3 isoforms exhibited no suppressive activity, and indeed moderately activated the Hes1 promoter (**Fig 5B**). In addition, the short Helios isoforms were able to revert the suppressive activity of Helios-1 and Ikaros-1 in a dose-dependent manner (**Fig 5C**). The above results demonstrate that Helios short variants exhibit a dominant-negative function in the regulation of transcriptional suppression by Helios-1 or Ikaros-1.

### Leukemia-type short Helios variants enhance T cell proliferation

**Fig 6:**
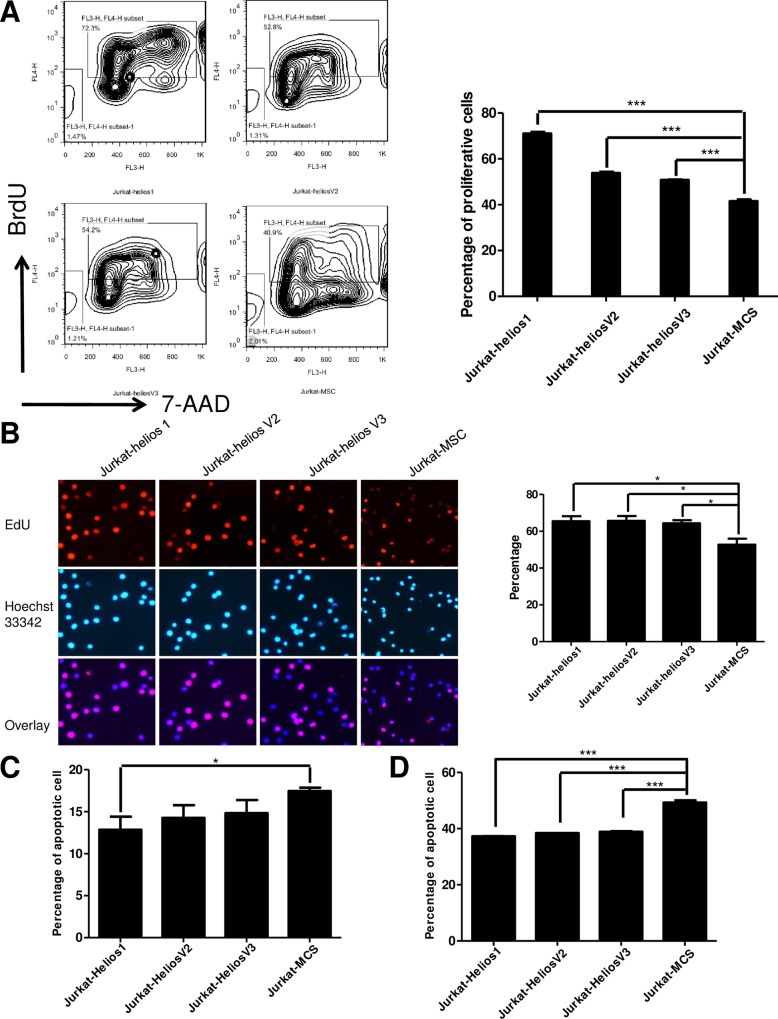
Short Helios variants function in T cell proliferation and survival. (**A**) Cell proliferation analysis of Helios-expressing Jurkat cells was examined by BrdU incorporation. The Jurkat cells stably expressing Helios variants were starved with no-serum media for 40 hr, and pulse-labeled with BrdU for 3 hr. Proliferation was determined by BrdU incorporation. Mean ± SD is shown (*** p< 0.05). (**B**) EdU assay of relative Hoechst33342 stained cells and EdU add-in cells. The proliferating capability of Helios-expressing Jurkat cells was evaluated using EdU incorporation. (*p<0.05) (**C**, **D**) The overexpression of Helios-V2 and–V3 protects against apoptosis in the presence of Cisplatin. Jurkat cells stably expressing Helios variants were evaluated for apoptosis by measurement of Annexin V staining (**C**) (*p<0.05) or evaluated with the treatment of Cisplatin (**D**). The data shown are representative of three different experiments. (***p<0.05)

Several studies have indicated that Ikaros acts as a tumor suppressor gene, and the overexpression of dominant-negative Ikaros isoforms 6 reduced the growth-inhibitory activity of Ikaros [11, 41, 42]. Therefore, we tested whether or not the novel abnormal splicing of Helios was capable of promoting T-cell leukemogenesis. In order to gain insight into the dominant-negative effects of the short Helios isoforms in T-cell growth of hematopoietic lineage cells, we established Jurkat cell lines that stably expressed the Helios-1, Helios-V2 and -V3 variants **(S2 Fig).** Cell proliferation assay by BrdU (**Fig 6A**) incorporation confirmed that the expression of these three variants significantly accelerated Jurkat cell proliferation, compared with the control cells. This result was confirmed by EdU cell proliferation assay (**Fig 6B**). EdU-positive cells significantly increased in the Helios 1 (65.4%), Helios-V2 (65.6%) and Helios-V3 cells (64.4%), compared with those in the control cells (52.7%). The ability of Helios isoforms to confer cell viability was confirmed by the Celltiter-Glo luminescent cell viability assay (Promega) (**S3A Fig**). Together, these data indicate that dominant-negative Helios isoforms contributed to T-cell growth.

Previous report indicated that Ikaros is involved in the control of apoptosis [9]. Overexpression of full-length Ikaros induced the increased apoptosis in leukemic cell lines [43]. In contrast, the overexpression of Ikaros 6 could delay apoptotic cell death, and rescue B lymphocytes from anti-IgM-induced apoptosis [44]. Thus, we tested the function of short Helios variants in T-cell apoptosis. The results showed that, the overexpression of Helios-V2 and -V3 did not significantly change the apoptotic rate of the Jurkat cells (**Fig 6C** and **S3B Fig**). Worth noting is that both Helios 1 and short variants Helios-V2 and -V3 protected Jurkat cells against cisplatin-induced apoptosis (**Fig 6D** and **S3C Fig**). Therefore, the expression of Helios alternative splicing isoforms controls both cell proliferation and cell death, thus indicating that these novel dominant-negative Helios isoforms play a potential role in leukemogenesis.

### The expression of Helios alternative splicing isoforms causes deregulation of various genes in T-cells

**Fig 7:**
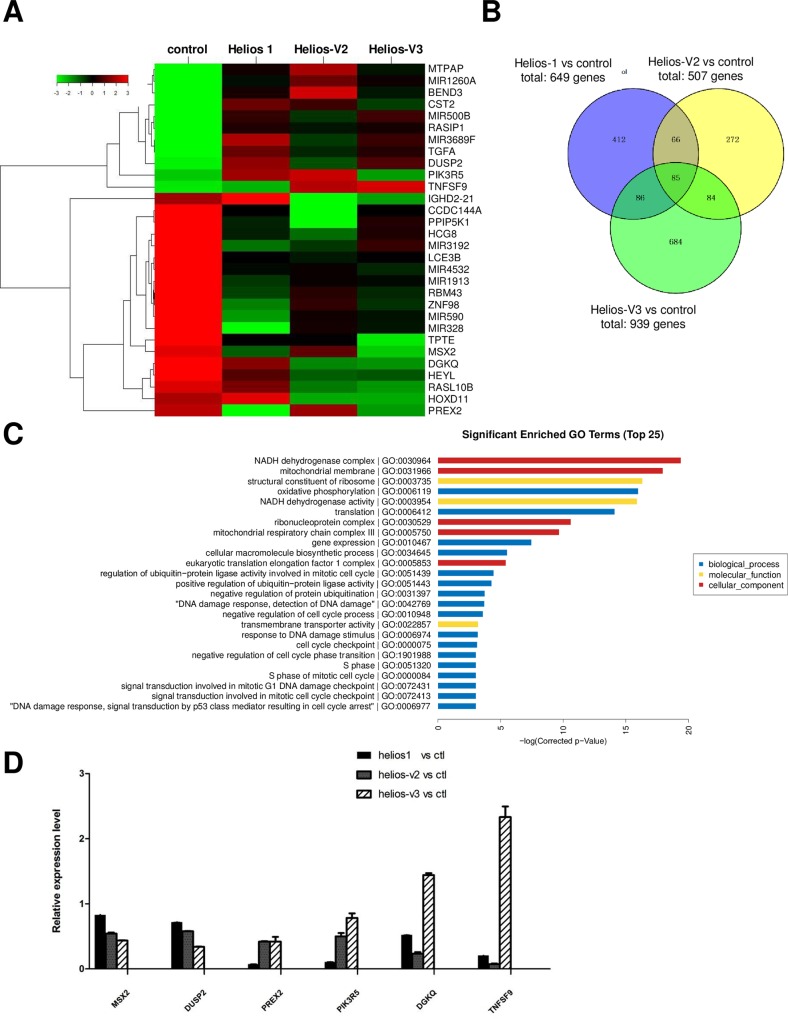
In-depth search of Helios targets by microarray analysis. (**A**) Hierarchical clustering of deregulated genes between Jurkat stable cells. Jurkat cells stably expressing Helios-1, and short variants Helios-V2, and Helios-V3 were used to analyze their gene expression patterns by the microarray. The 2D hierarchical clusters between the cells expressing Helios variants are indicated. (**B**) Venn diagram showing numbers of genes differentially expressed in the Jurkat stable cell lines versus control. The differential expression gene set (1.5-fold changes) was compared. (**C**) Pathway analysis of genes deregulated in Helio-V3 Jurkat cells relative to their expression in control cells. Number along vertical line indicates *P* value (-log) for pathway discovery. (**D**) Analysis of relative gene expression by quantitative RT-PCR in Jurkat cells stably expressing Helios splice variants compared with mock transfected Jurkat cells.

As a member of a family of hematopoietic-lineage transcription factors, it is expected that the expressions of dominant-negative Helios variants leads to the alteration in the expression pattern of various downstream genes compared to full-length Helios 1. In order to verify this, gene expression profiling was performed on Jurkat cells stably expressing Helios-1, as well as short variants Helios-V2 and Helios-V3. With a > 1.5-fold cutoff, the number of differentially expressed genes was higher in the Helios-V3 Jurkat cells versus the control (n = 939), than in the Helios 1 Jurkat cells versus the control (n = 649), or the Helios-V2 Jurkat cells versus the control (n = 507) (**Fig 7A and 7B**). Among these genes are known candidate oncogenes or tumor suppressors (WNT3, TP53, pim-1, JUN, EGR1, TGFA). Some target genes are involved in leukemic translocation (MLLT4, TCF3 fusion partner). Some candidate targets (RUNX3, IRF8, EBF3, PTCH1, ID2, C/EBP zeta, GATA2) function in hematopoietic lineage commitment and differentiation. Worth noting is that Notch signaling pathways (Jagged-2, Notch2NL, HEYL, HES6, HEY2) were deregulated between the transformants and control cells. The genes involved in hematopoietic-lineage markers (CD7, CD24, CD40LG, CD34, CD69, granzyme A, TNFSF9) were among the deregulated genes. Ras family proteins (HRAS, RASA4, RASIP1, RASL10B, RalB, RAB33A), serine/threonine-protein kinases (SYK, SKAP1, VRK2, RIPK4) and components of inositol kinase pathways (PPIP5K1, PIK3R5, DGKQ) were among the differentially expressed genes. Transcriptional machinery genes (EEF1A2, EEF1B2, EEF1G, H3 histone), cell-cycle genes (CDKN2D, CDC26) and apoptosis pathways (BAX, BCL2L12) were also found to be deregulated. In addition, Helios isoforms appeared to regulate the expression of major histocompatibility complex (HLA-E, HLA-C).

Furthermore, GO pathway analysis [45] of deregulated gene set in Helios-V3 transformed Jurkat cells identified the enrichment of several signaling cascades (**Fig 7C**). These pathways are essential for T-cell development and regulations, e.g. translation, gene expression, cellular macromolecule biosynthetic process, translational elongation, ribosome biogenesis, and DNA damage response. Among these pathways, it has not been reported that the pathways of the regulation of cell cycle process and checkpoint are regulated by the Helios family. Furthermore, we confirmed the overexpressed PIK3R5, DGKQ and TNFSF9, which are crucial components of kinase pathways involved in leukemia and lymphoma, in manipulated Jurkat samples (**Fig 7D**).

## Discussion

It is well known that some human genes diversify their encoded proteins by means of alternative splicing. These proteins produced by alternative splicing can be changed either subtly or dramatically, by means of eliminating regions utilized in interactions with their partners or sub-cellular localizations [46, 47]. The Ikaros family members, including Helios, are master transcription factors of hematopoietic commitment and differentiation, and manifest multiple isoforms [40]. The presence of diversified, alternatively spliced Ikaros family proteins in hematopoietic lineage cells raises some interesting questions with respect to their function and the modulation of their biological activities. In addition, the capability of Ikaros gene-family members to form homo- or heterodimers increases the complexity of this topic.

In this study, we reported the identification of three novel Helios dominant-negative isoforms, respectively named Helios-V1, -V2 and -V3. Among them, Helios-V1 protein has all six zinc-finger motifs, but lacks exon 6 for nuclear entry. Helios-V2 and -V3 are non-canonical isoforms which lack four N-terminal zinc-finger motifs, thereby absent of the functional DNA-binding domain. Interestingly, although Helios-V2 and -V3 donot have C-terminal dimerization domain, they are capable of relocating the nuclear localization of Ikaros into cytoplasm, modifying the gene transcription of the target gene, and consequently functioning as dominant-negative isoforms. It is possible that the homologous N-terminal region can impact on the tertiary folding, stability and phosphorylation acceptor sites of the Helios protein structure, and thereby can mediate the interaction by binding and inducing the cytoplasmic sequestration of the Ikaros-family genes [48]. In addition, we showed a novel molecular feature of leukemia-type cells, which have profound abnormal expression of transcription factor Helios. The abnormal alternative splicing seemed to participate in the advantageous cell survival and growth in leukemic cells. Our results also demonstrated the tumor-suppressive abilities and downstream targets, as well as the pathways of Helios isoforms, in human leukemic T-cell lines.

The analysis of Ikaros family members highlighted profound abnormalities in Helios expression in leukemia and lymphomas. In our study, the expression patterns of Helios alternative splicing were characterized in cells originated from different lymphoid malignancies. These analyses led us to isolate three novel Helios isoforms. Our results also indicated that short Helios variants were expressed in PBMCs of leukemia patients, which suggest that the aberrant splicing of Helios may be present in leukemia development. The structures of leukemic-type Helios variants are characterized by a selective absence of four zinc fingers in the N-terminal DNA-binding domain, or exon 6 which possibly contains a nuclear entry signal. Our findings suggested that these isoforms lacked DNA binding activity or the ability of nuclear entry, although the capability of dimerization was preserved. Thus, these alternative splice isoforms interfered with the transcriptional regulations of the Ikaros family proteins, and accordingly exhibited dominant-negative activities. In addition, our results showed that the overexpression of Helios short variants enhanced cell proliferation by BrdU and EdU incorporation, and strongly protected Jurkat cells from cisplatin-induced apoptosis. Taken together, these results indicate that the alterations of Helios expression, including the lack of or diminished Helios-1 expression and upregulated expression of Helios alternative short variants, result in the abrogation of tumor-suppressor functions of Ikaros gene family in leukemic cells.

For these reasons, we examined the unique features of these different isoforms modulated by different combinations of functional domains, including subcellular localization, interaction with Ikaros and components of HDAC complexes, transcriptional regulation of putative target genes, and the survival and proliferation properties when overexpressed in T-cell lines. We propose that the short isoforms which are absent of certain domains and exhibit dominant-negative functions could interact with full-length isoforms and modulate their activity. For example, the co-expression of Helios-V1, -V2 or -V3 with full-length Ikaros isoform (Ikaros-1) leads to a switch of sub-cellular distribution of Ikaros from nuclei to cytosol, most likely as a result of heterodimerization.

In addition, there is an interesting difference regarding the cellular distribution of full-length Helios-1 and Helios-V1, which only differ in exon 6 (48 amino acids) and result in the change of nuclear localization to the cytosol. Consistent with previous research, this suggests the presence of a nuclear localization signal in exon 6 [4]. Helios-V2 and -V3, two novel isoforms described in this report, originate from alternative splicing with the change of the open reading frame of exon 7, and generate the nonsense mutation. These two isoforms exhibit nuclear and cytoplasmic localization and interact with HDAC1 and MTA2, as a component of Mi-2β/NuRD histone deacetylase complexes. Although these two short isoforms lack the C-terminal dimerization domain, they can still interact with Ikaros-1 by changing its cellular distribution, and modulate the transcription activity of Hes-1 when Ikaros-1 or Helios-1 was co-transfected by dual-luciferase assays. In addition, these two isoforms, which lack the N-terminal four zinc finger motifs of DNA-binding domain, may quench a fraction of Helios-1 or Ikaros-1 by dominant-negative activity, and thus reduce the activity of full-length Ikaros as tumor suppressor. This fact was demonstrated by the increased proliferation of Jurkat cells stably transfected with Helios-V2 and -V3.

We also confirmed that *foxp3* which is master transcription factor in regulatory T cells, and *Hes1*, a target gene of the Notch1 signaling pathway, are targets of Helios. Recently, a study suggested that the activation of the Notch1 signaling pathway may contribute to adult T-cell leukemia and that Hes1 is upregulated in leukemic samples [49]. Therefore, we examined the transcription activity of Helios short variants by means of dual-luciferase reporter assay and confirmed the regulation. It was shown that Hes1 promotes cell proliferation directly by the transcriptional repression of p27kip1 [50]. Thus, our findings indicate that abnormal Helios expression is possibly the initiating element of Hes1 activation, and is responsible for the genetic events that lead to T-cell leukemogenesis.

Our results indicate that the short Helios variants (V1-V3) may act as an oncogene. In comparison, wild-type Helios (Helios-1, and -2) exhibits tumor suppressor-like activity. Consistently, earlier research has identified that the expression of a non-DNA-binding isoform of Helios is lymphomagenic in chimeric mice [25]. In addition, our analysis of the gene expression profiles of the Jurkat cells stably transformed with various Helios isoforms showed the activation of important pathways involved in gene expression, translation, RNA processing, cell cycle and DNA damage response.

In summary, our results identified the deregulation in Helios alternative splicing as a novel perspective of molecular abnormalities in leukemia and lymphoma, which seems to function importantly in T-cell proliferation and the regulation of gene expression. Additionally, the data presented here provide the insight that the subtle modifications in the expression of dominant-negative isoforms result in significant differences in Helios or Ikaros function, thereby revealing Helios alternative variants as targets for therapeutic intervention in lymphocyte malignancies.

## Supporting Information

S1 Fig Amino acid sequence alignment of Helios short variants and Ikaros 1. Alignment of the amino-acid sequence of the three Helios short variants with the sequence of the canonical full-length isoforms of the Helios 1 and Helios 2, and Ikaros 1 is shown. The filled blue boxes indicate the conserved zinc finger motifs. Yellow bars emphasize the highly conserved cysteines and histidines within the zinc finger motifs. The conserved transcriptional activation domain is shown in green line [19].
(TIF)

S2 Fig Establishment of Jurkat cell lines stably expressing full-length Helios-1, and short variants Helios-V2 and–V3. The Helios expression was confirmed by immunoblotting with anti-Flag Ab.
(TIF)

S3 Fig Helios isoforms confer the increased cell viability and the resistance to cisplatin-induced apoptosis. (**A**) Jurkat cell lines stably expressing Helios isoforms were subjected to celltiter-glo luminescent cell viability assay and the results were read by luminescent output. (**B, C**) Apoptosis profiles of Helios-expressing Jurkat cell lines indicated by Annexin V and PI (**B**), or when subjected by cisplatin treatment (**C**).
(TIF)
