# Supplementary figures and images for: Alternative Splice Variants Modulates Dominant-Negative Function of Helios in T-Cell Leukemia
Source: PLoS One. 2016 Sep 28;11(9):e0163328. doi: 10.1371/journal.pone.0163328 (PMC5040427; doi:10.1371/journal.pone.0163328)

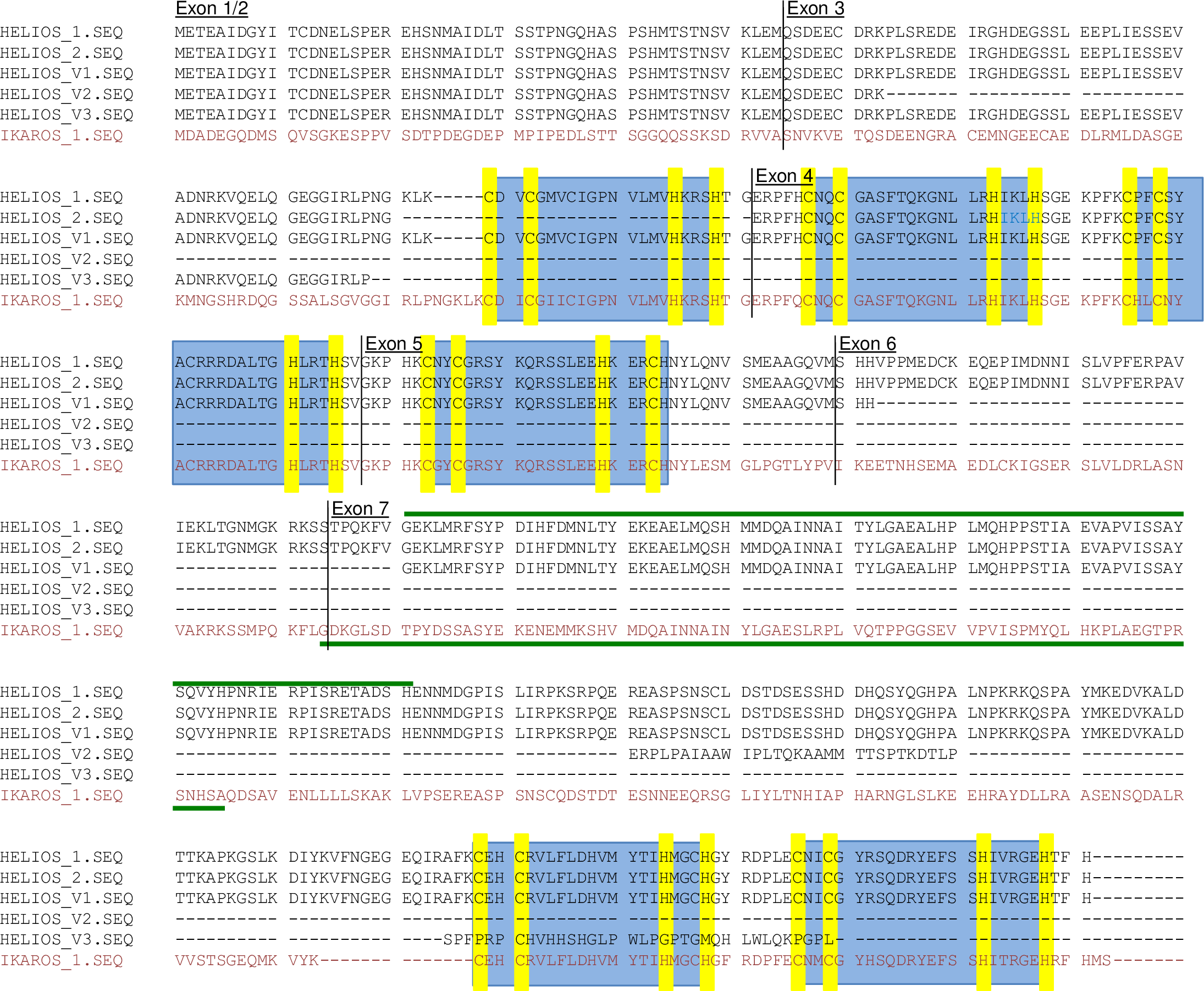

Supplement: S1 Fig — Alignment of the amino-acid sequence of the three Helios short variants with the sequence of the canonical full-length isoforms of the Helios 1 and Helios 2, and Ikaros 1 is shown. The filled blue boxes indicate the conserved zinc finger motifs. Yellow bars emphasize the highly conserved cysteines and histidines within the zinc finger motifs. The conserved transcriptional activation domain is shown in green line [19]. (TIF) [file pone.0163328.s001.tif]

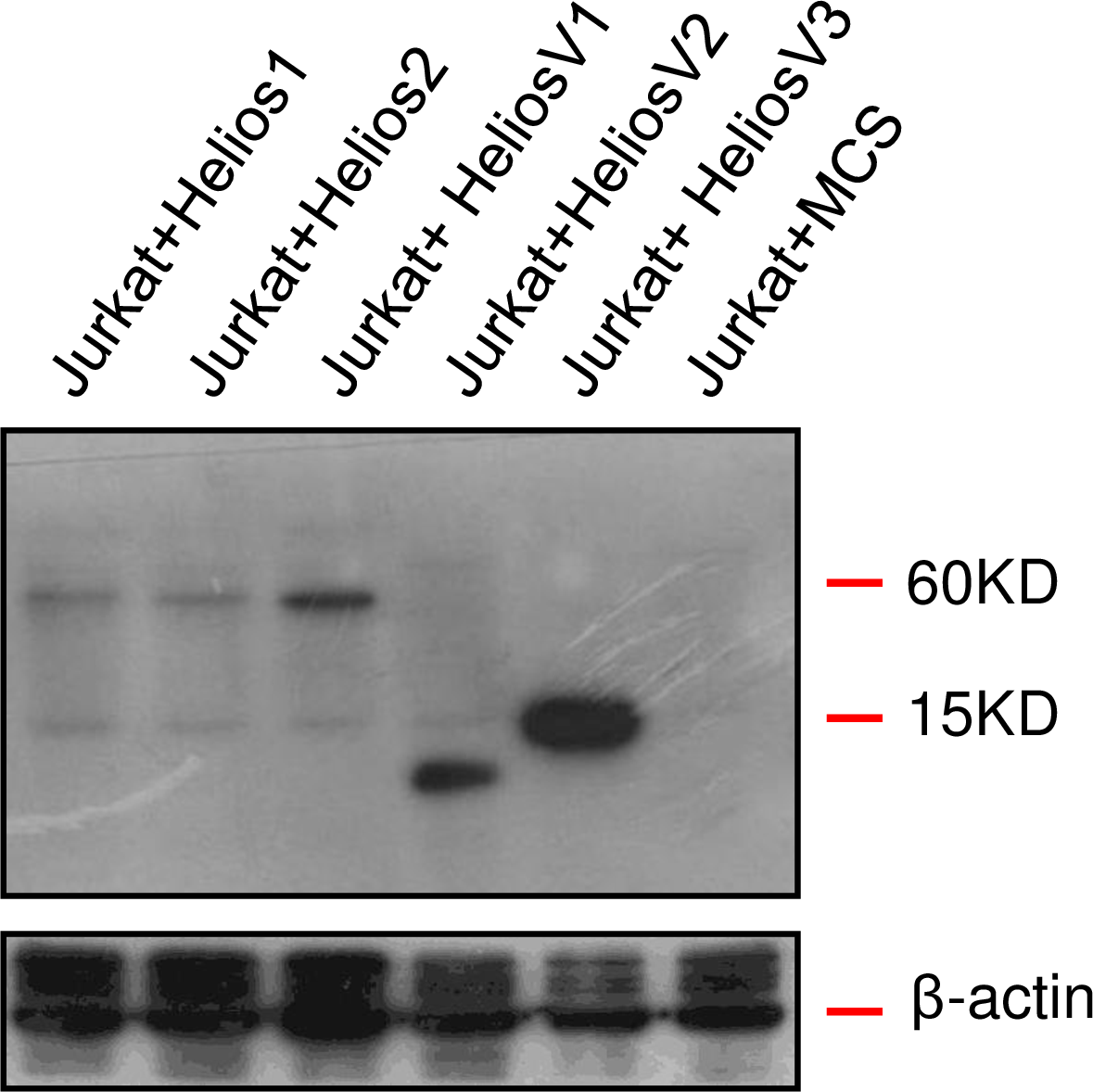

Supplement: S2 Fig — The Helios expression was confirmed by immunoblotting with anti-Flag Ab. (TIF) [file pone.0163328.s002.tif]

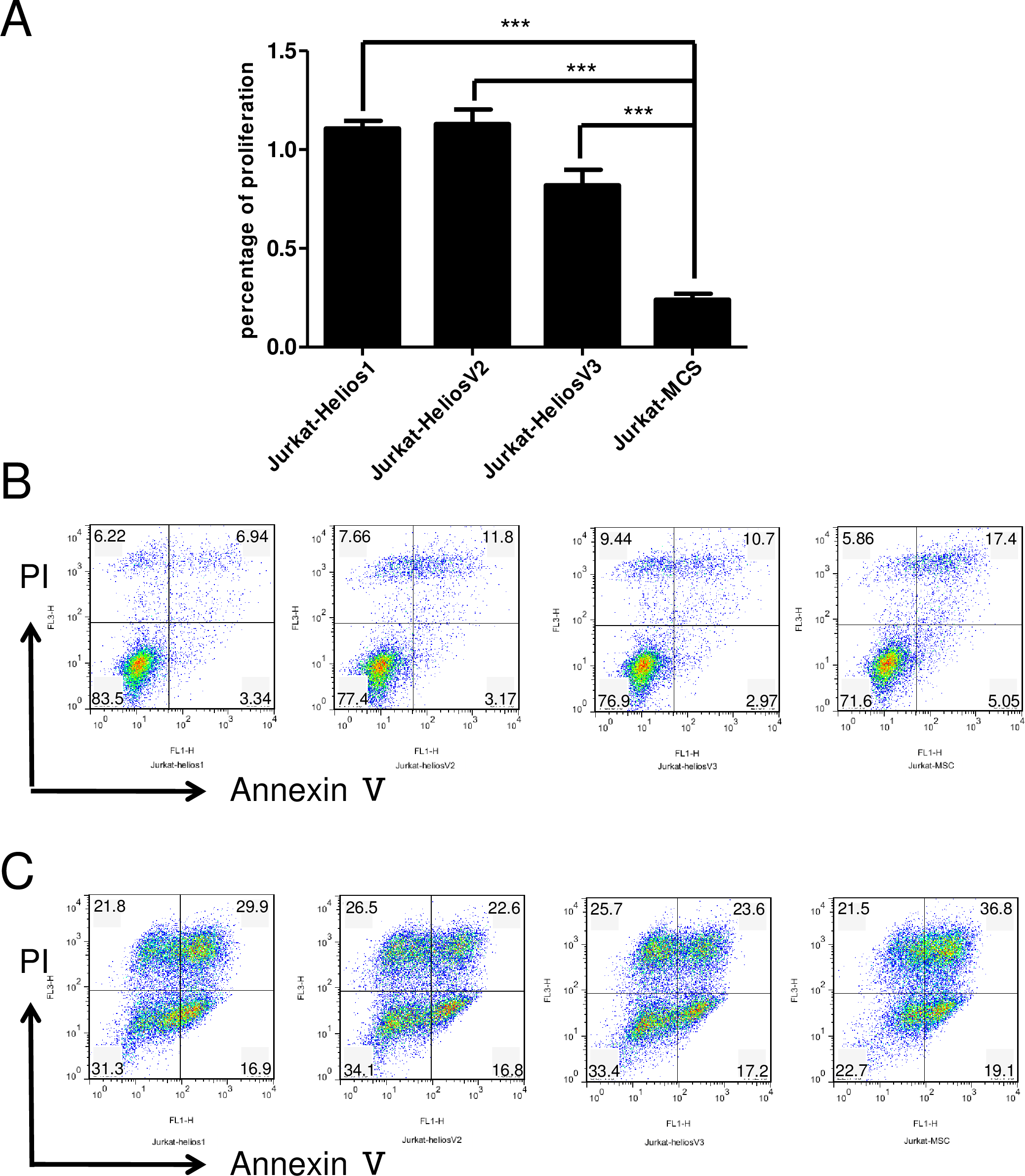

Supplement: S3 Fig — (A) Jurkat cell lines stably expressing Helios isoforms were subjected to celltiter-glo luminescent cell viability assay and the results were read by luminescent output. (B, C) Apoptosis profiles of Helios-expressing Jurkat cell lines indicated by Annexin V and PI (B), or when subjected by cisplatin treatment (C). (TIF) [file pone.0163328.s003.tif]
